# Human skeletal muscle is refractory to the anabolic effects of leucine during the postprandial muscle-full period in older men

**DOI:** 10.1042/CS20171230

**Published:** 2017-10-27

**Authors:** W. Kyle Mitchell, Bethan E. Phillips, Ian Hill, Paul Greenhaff, Jonathan N. Lund, John P. Williams, Debbie Rankin, Daniel J. Wilkinson, Kenneth Smith, Philip J. Atherton

**Affiliations:** 1 MRC-ARUK Centre for Musculoskeletal Ageing Research and NIHR Nottingham BRC, School of Medicine, Royal Derby Hospital, University of Nottingham, U.K.; 2 Department of Surgery, School of Medicine, Royal Derby Hospital, University of Nottingham, U.K.; 3 MRC/ARUK Centre for Musculoskeletal Ageing Research and NIHR Nottingham BRC, School of Life Sciences, Queen’s Medical Centre, Nottingham, U.K.

**Keywords:** amino acid metabolism, mechanistic target of rapamycin, nutrition, protein synthesis, skeletal muscle

## Abstract

Leucine modulates muscle protein synthesis (MPS), with potential to facilitate accrual/maintenance of muscle mass. Animal models suggest that leucine boluses shortly after meals may prolong MPS and delay onset of a “muscle-full” state. However, the effects of nutrient “top-ups” in humans, and particularly older adults where deficits exist, have not been explored. We determined the effects of a leucine top-up after essential amino acid (EAA) feeding on anabolic signaling, MPS, and muscle energy metabolism in older men. During ^13^C_6_-phenylalanine infusion, 16 men (∼70 years) consumed 15 g of EAA with (*n*=8, FED + LEU) or without (*n*=8, FED) 3 g of leucine top-up 90 min later. Repeated blood and muscle sampling permitted measurement of fasting and postprandial plasma EAA, insulin, anabolic signaling including mTOR complex 1 (mTORC1) substrates, cellular ATP and phosphorylocreatine, and MPS. Oral EAA achieved rapid insulinemia (12.5 iU·ml^−1^ 25 min post-feed), essential aminoacidemia (3000 μM, 45–65 min post-feed), and activation of mTORC1 signaling. Leucine top-up prolonged plasma EAA (2800 μM, 135 min) and leucine availability (1050 μM, 135 min post-feed). Fasting FSRs of 0.046 and 0.056%·h^-1^ (FED and FED + LEU respectively) increased to 0.085 and 0.085%·h^-1^ 90–180 min post-feed and returned to basal rates after 180 min in both groups. Phosphorylation of mTORC1 substrates returned to fasting levels 240 min post-feed in both groups. Feeding had limited effect on muscle high-energy phosphates, but did induce eukaryotic elongation factor 2 (eEF2) phosphorylation. We demonstrate the refractoriness of muscle to nutrient-led anabolic stimulation in the postprandial period; thus, leucine supplements should be taken outside of meals, or with meals containing suboptimal protein in terms of either amount or EAA composition.

## Introduction

The increase in plasma amino acid (AA) concentration that follows ingestion, digestion, and absorption of dietary protein is the major anabolic drive contributing to the reversal of the postabsorptive net efflux of amino acids from human skeletal muscle—shifting the dynamic equilibrium toward net accretion of myofibrillar mass and replacing losses since the last meal [1]. A dose–response relationship exists such that although there is a graded response to small feeds [2], a modest serving of essential amino acids (EAA) or high quality protein (i.e. rich in EAA) achieves the same response as a large serving [3,4]. Recent advances in stable isotope tracer techniques have permitted detailed temporal resolution of muscle metabolic responses to EAA/protein feeding. These techniques have made it apparent that after an initial latent period (~30 min for intravenous EAA infusion, ~45 min for whey, and up to 90 min for free EAAs), muscle protein synthesis (MPS) in healthy young humans approximately doubles for a finite period (∼2–3 h) before returning to fasted rates [5–7]. The observation that MPS returns to fasting rates despite ongoing availability of EAA and activity within the mTOR complex 1 (mTORC1) signaling pathway [5] suggests a so-called “muscle-full” effect. It is likely that the underlying mechanisms regulating onset of the muscle-full state will depend upon accretion of adequate new muscle protein rather than being simply time-dependent, since the onset of muscle-full appears to be delayed with the provision of a very gradual aminoacidemia (achieved via comparing pulse with bolus EAA) feeding [8].

Individual amino acids have distinct potencies as muscle anabolic stimulants. Only the EAA stimulate increases in MPS in humans [9–11] and among these, branched-chain amino acids (BCAAs) are distinct in being preferentially metabolized in skeletal muscle rather than gut or liver [12–14]. Of the BCAAs, leucine is unique in directly activating the mTORC1 protein kinase, a master growth controller [15,16], promoting AA uptake by peripheral tissue [17] and maximizing MPS [18,19]; this underpins the academic [20] and commercial [21] interest in its role as a pharmaconutrient. Studies in rats have shown that while the leucine content of a mixed meal directs the peak activation (but not duration) of MPS [22] the provision of leucine in the postprandial period (120 min post-feed) can reinvigorate the maximal stimulation of MPS as it is waning, i.e. delaying the onset of muscle-full [23]. As such, this demands a better understanding of the muscle-full state in humans including assessment of the refractoriness of skeletal muscle in the postprandial/early postabsorptive period to further anabolic stimulation, which has not, to our knowledge, been explored. Moreover, given anabolic resistance to feeding in older age [2] and over which contention remains as to whether this can [24,25] or cannot [2] be overcome by increasing the dosage, this is a particularly relevant question in older people.

The purpose of the present study was to assess the refractoriness of older human skeletal muscle to anabolic stimulation when in the muscle-full state, by provision of 3 g of oral leucine bolus or “top-up” 90 min after a 15 g of mixed EAA feed, and to contextualize this response in terms of activity within established anabolic signaling pathways and cellular energy stress markers, within muscle cells. This work provides new insight into both fundamental nutritional biochemistry and applied nutrition.

## Experimental detail

### Study design

**Figure 1:**
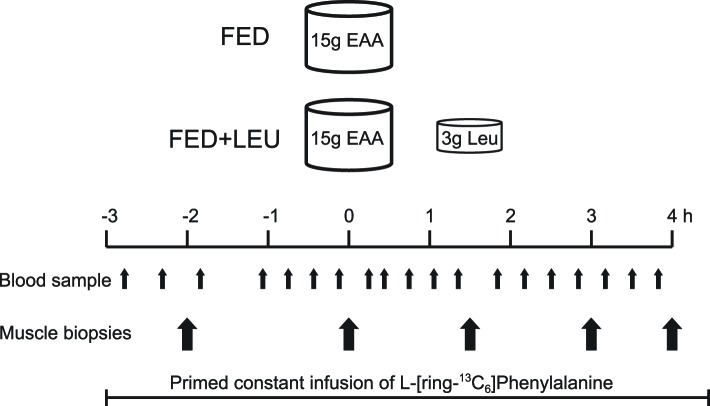
Experimental protocol During a constant primed infusion of [^13^C_6_]-phenylalanine, 15 g of essential amino acids were ingested with or without a 3 g of leucine top-up 90 min later. Blood samples and muscle biopsies allowed assessment of plasma amino acid and insulin concentrations, fractional synthetic rate of myofibrillar proteins, signaling pathways, and muscle energy metabolism; EAA, essential amino acid; Leu, leucine.

With ethical approval from the University of Nottingham Medical School Ethics Committee (U.K.), this work was carried out in accordance with the Declaration of Helsinki, with prospective registration (clinicaltials.gov registration no. NCT01890369). Healthy, recreationally active older males (*n*=16, 70.3 ± 2.6 years, BMI 25.5 ± 1.8 [Mean ± SD]) were recruited by mail and local advertising. Recruits were studied after fasting overnight and were asked to avoid heavy exercise for the preceding 48 h. On the morning of the study (08.00), participants had a 18 g of cannula inserted into the dorsum of the left hand for a primed (0.4 mg·kg^−1^) constant infusion (0.6 mg·kg^−1^ ·h^−1^) of L-[ring-^13^C_6_]-phenylalanine (Cambridge Isotopes Ltd., Cambridge, MA, U.S.A.) tracer. Blood samples and muscle biopsies were taken according to the protocol (Figure 1). Arterialized venous blood was sampled via a retrograde 16 g of intravenous cannula placed in the dorsum of the right hand, with the hand warmed to 55°C [26]. Muscle biopsies were taken intermittently from m. vastus lateralis using the conchotome technique [27] after infiltration of 5 ml of 1% lignocaine. Muscle was washed in ice-cold saline and visible fat and connective tissue were removed before being frozen in liquid N_2_ and stored at −80°C until analysis. Biopsies were taken 1 and 3 h after commencement of tracer to permit assessment of basal (postabsorptive) MPS. Participants were then provided with 15 g of mixed essential amino acids (histidine, 1.21 g; isoleucine, 1.73 g; leucine, 3.59 g; lysine, 3.07 g; methionine, 0.95 g; phenylalanine, 0.91; threonine, 1.13 g; tryptophan, 0.48g; and valine, 1.86 g) in aqueous solution (200 ml), which was consumed in a single draft. Participants were allocated to receive just this EAA bolus feed (FED, *N*=8) or this feed and an additional leucine “top-up” of 3 g in aqueous solution (200 ml) consumed 90 min after the initial EAA feed (FED + LEU, *N*=8). Subsequent biopsies at 90, 180, and 240 min post-commencement of feeding allowed assessment of MPS in the intervening periods. After the study, cannulae were removed and the participants fed and monitored for 30 min before departure. Subject demographics are shown in Table 1.

**Table 1:** Volunteer characteristics, values are means ± standard deviations

	EAA	EAA + LEU	*P* value
Age, years	70.0 ± 2.3	70.6 ± 3.0	0.67
Height, cm	172 ± 6	176 ± 5	0.18
Weight, kg	75.4 ± 5.5	78.7 ± 8.2	0.37
BMI, kg·m^−2^	25.6 ± 1.1	25.5 ± 2.4	0.96
ASMMI, kg·m^−2^	8.16 ± 0.47	7.83 ± 0.87	0.36
HGS, kg	40.7 ± 3.5	42.4 ± 8.5	0.64

Abbreviations: ASMMI, appendicular skeletal muscle mass index (appendicular muscle mass·height^−2^); HGS, hand grip strength.

### Measurement of plasma AA and insulin concentration

Venous plasma insulin concentrations were measured using undiluted samples on a high-sensitivity human insulin enzyme-linked immunosorbent assay (DRG Instruments GmbH, Marburg, Germany). Area under the curve (AUC) analysis estimated the total insulin response to feeding and was calculated for each individual with a baseline equal to the mean of fasting, +155 min and +195 min insulin concentrations.

For AA analyses, equal volumes of arterialized plasma and 10% sulfosalicyclic acid were mixed and cooled to 4°C for 30 min. Samples were centrifuged at 8000 ***g*** to pellet the precipitated protein and the supernatant fluid was passed through a 0.22 µm filter before analysis with a dedicated AA analyzer, based on ion exchange chromatography, using lithium buffers with post column derivatization with ninhydrin (Biochrom 30, Biochrom, Cambridge, U.K.) using lithium buffers. All 20 AA concentrations were measured by comparison with a standard AA mix with norleucine as an internal standard. AUC analysis estimating total EAA exposure was calculated for each individual without baseline correction.

### Measurement of myofibrillar protein fractional synthetic rate

The fractional synthetic rate (FSR) of myofibrillar protein was derived from the increase in incorporation of L-[ring-^13^C_6_]-phenylalanine between subsequent muscle biopsies. Muscle intracellular phenylalanine, the average of two biopsies, was used as a surrogate of phenylalanyl-tRNA labeling (the immediate precursor for protein synthesis) [28]. The standard precursor–product method was used to calculate FSR (%·h^-1^).

$$\text{FSR}=\left[\frac{E_{\text{m}2}-E_{\text{m}1}}{E_{\text{p}}\;\times \;t}\right]\;\times \;60\;\times \;100$$

where *E*_m1_ and *E*_m2_ are the enrichments of bound L-[ring-^13^C_6_]- phenylalanine in two sequential biopsies, *t* is the time interval between two biopsies, and *E*_p_ is the mean L-[ring-^13^C_6_]-free phenylalanine enrichment in the intramuscular pool.

To maintain steady-state labeling, the potential dilution of enrichment in plasma (and, in turn, intramuscular) phenylalanine labeling with EAA feeding was offset by provision of 6% of ingested phenylalanine as L-[ring-^13^C_6_]- phenylalanine.

In relation to quality control (QC) for mass spectrometry (and amino acid analyses), we constructed both concentration and enrichment curves and ran QC samples alongside each batch of analyses for both plasma and intramuscular Phe by GC–MS—in addition to enrichment curves and QC samples (which bracket the expected enrichment of the myofibrillar protein bound ^13^C_6_ Phe) for the GC–C–IRMS analysis. Repeat analyses have coefficients of variation (CV) of <5% and standard curves have *R*^2^ greater than 0.995. All equipment is subject to regular maintenance to maintain specification.

### Measurement of intramuscular high-energy phsophates

Approximately 10 mg of frozen muscle was cut from each biopsy, freeze-dried, and stored at −80°C for subsequent muscle metabolite analysis. Freeze-dried muscle samples were powdered, after removal of macroscopic blood and connective tissue, and metabolites extracted in 0.5 M perchloric acid (containing 1 mM EDTA), followed by neutralization with 2.2 M K_2_CO_3_ (Sigma Chemicals). Muscle ATP, phosphorylocreatine (PCr), and free creatine concentrations were determined enzymatically in muscle extracts according to the method of Harris et al. [29], which was modified to accommodate the use of a 96-well plate spectrophotometer. Muscle total creatine was calculated as the sum of PCr and creatine and subsequently used to correct muscle ATP and PCr values for nonmuscle constituents.

Immunoblotting was performed on ~30 mg of muscle as described previously [18]. Primary antibodies against phospho-ribosomal protein 70 S6 kinase 1 (p70 S6K) Thr398, protein kinase B (AKT) Ser473, eukaryotic elongation factor 2 (eEF2) Thr56, eukaryotic initiation factor 2α (eIF2α) Ser51, acetyl Co-A carboxylase (ACC) Ser79, AMP-activated protein kinase (AMPK) Thr173, (New England Biolabs), and eukaryotic translation initiation factor 4E-binding protein 1 (4EBP1) Ser65/Thr70 (Santa Cruz Biotechnology) were used before incubated in HRP-conjugated secondary antibody (New England Biolabs). Membranes were exposed to chemiluminescent HRP substrate (Millipore) and bands were quantified using Chemidoc XRS (BioRad). Protein loading anomalies were normalized to Coomassie-stained membranes and each individual was standardized to fasting. Blot data were analyzed by using peak density and all signals remained within the linear range of detection, avoiding saturation. Immunoblots were run using prevalidated antibodies with specificity verified by positive and negative controls and, where required, peptide competition assays/*in vitro* siRNA/pharmacological ablation of signals for phospho-targets [30]. All samples were run alongside molecular weight standards to verify the expected molecular weight of target proteins.

### Statistical analyses

An *a priori* power calculation suggested we needed eight subjects per group to detect, with α of 0.05 and β of 0.85, a difference between groups of ∼20% in the primary end-point of MPS. Data are presented as means ± SE, after D’Agostino & Pearson omnibus normality testing. Demographic and anthropometric comparisons between groups were conducted by unpaired *t*-tests. Differences for all other analyses were detected by repeated-measures analysis of variance, with Bonferroni correction, using GraphPad Prism version 5 (GraphPad Software, San Diego, CA, U.S.A.). *P*<0.05 was considered significant, with two-tailed testing to compare between groups and one-tailed tests to detect changes from fasting. Effect size is presented for key comparisons between groups. For a given time point, this is estimated as ([mean of FED] − [mean of FED + LEU]) × (standard deviation of FED)^−1^.

## Results

### Plasma concentrations of AAs and insulin

**Figure 2:**
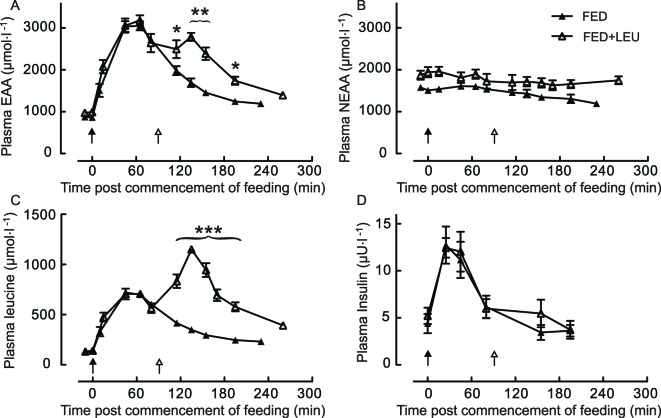
Amino acid and insulin responses to feeding. Plasma essential amino acids (EAA; **A**), nonessential amino acids (NEAA; **B**), leucine (**C**), and insulin (**D**) concentrations, in older men after consumption of 15 g of mixed EAA in the absence (FED) and presence (FED + LEU) of a 3 g of leucine top-up after 90 min; *P<0.05, **P<0.01, and ***P<0.001, differences between feeding strategies. Values are means + SE; *n=*8. Filled arrows represent ingestion of 15 g of EAA; unfilled arrows represent 3 g of leucine (EAA + LEU only).

After consumption of 15 g of mixed EAA, plasma EAA and leucine concentrations rise rapidly and by a similar amount in both FED and FED + LEU groups, peaking at between 45 and 65 min (EAA +320%, leucine +430%, all *P*<0.001 versus fasting) and falling thereafter. Provision of a 3 g of leucine top-up at 90 min after the initial 15 g of EAA feed provided a second peak in both plasma EAA (+190% from fasting, effect size 3.8) and leucine (+770% from fasting, effect size 12) concentration at 135 min post-initial feeding (45 min post top-up). Thus, plasma concentration of EAA and leucine differed between feeding regimens beyond 115 min (Figure 2A and 2B). Nonessential amino acid (NEAA) concentrations do not differ between groups and modulate little throughout the experimental protocol (Figure 2C). Both groups demonstrated a similar modest plasma insulin response, seen at 25 and 45 min (+160% at 25 min and +140% at 45 min, all *P*<0.001 versus fasting) and both groups had returned to fasting insulin concentrations at 80 min, with no subsequent divergence detected between groups (Figure 2D).

### Myofibrillar protein synthesis

**Figure 3:**
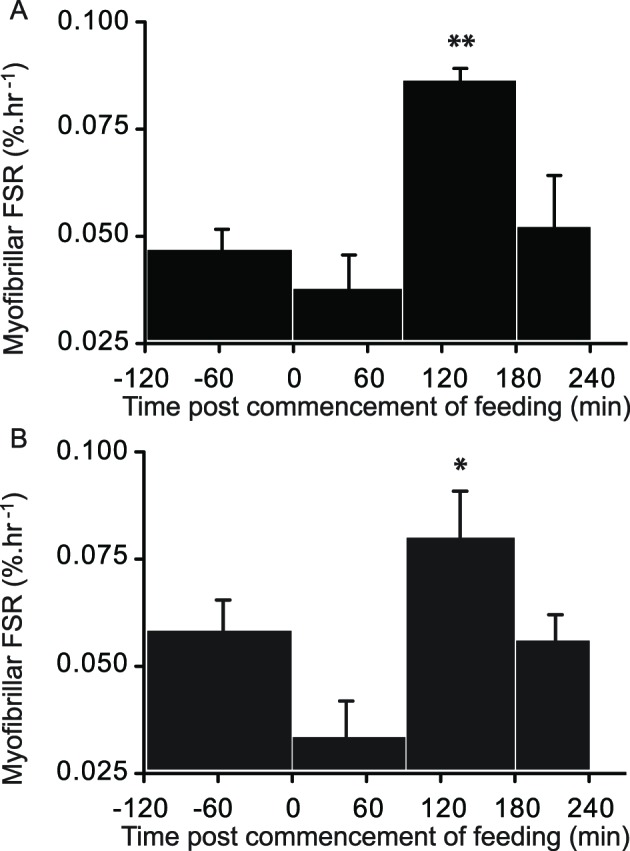
Myofibrillar protein synthesis. Fractional synthetic rate (FSR) of muscle protein synthesis in older men after consumption of 15 g of mixed EAA in the absence (**A**) and presence (**B**) of a 3 g of leucine top-up after 90 min. Values are means + SE, *n*=8; *P<0.05, **P<0.01, difference from fasting.

Fasting myofibrillar protein FSR was not different between groups (FED 0.046 ± 0.015% per h, FED + LEU 0.056 ± 0.018% per h, NS). After a latency of 90 min, FSR increased (FED 0.085 ± 0.003% per h and FED + LEU 0.085 ± 0.009% per h, *P*=0.031 and *P*=0.004, both versus fasting). Beyond 180 min, FSR returned to basal rate (FED 0.051 ± 0.012% per h, NS versus fasting) and the provision of a leucine top-up did not prolong stimulated FSR in the FED + LEU (0.055 ± 0.006% per h, NS versus fasting. The effect size of the addition of a leucine top-up, with regard to FSR between 180–240 min, was 0.12. Thus, no detectable difference in FSR existed between feeding regimens (Figure 3).

### Activity within established signaling pathways

**Figure 4:**
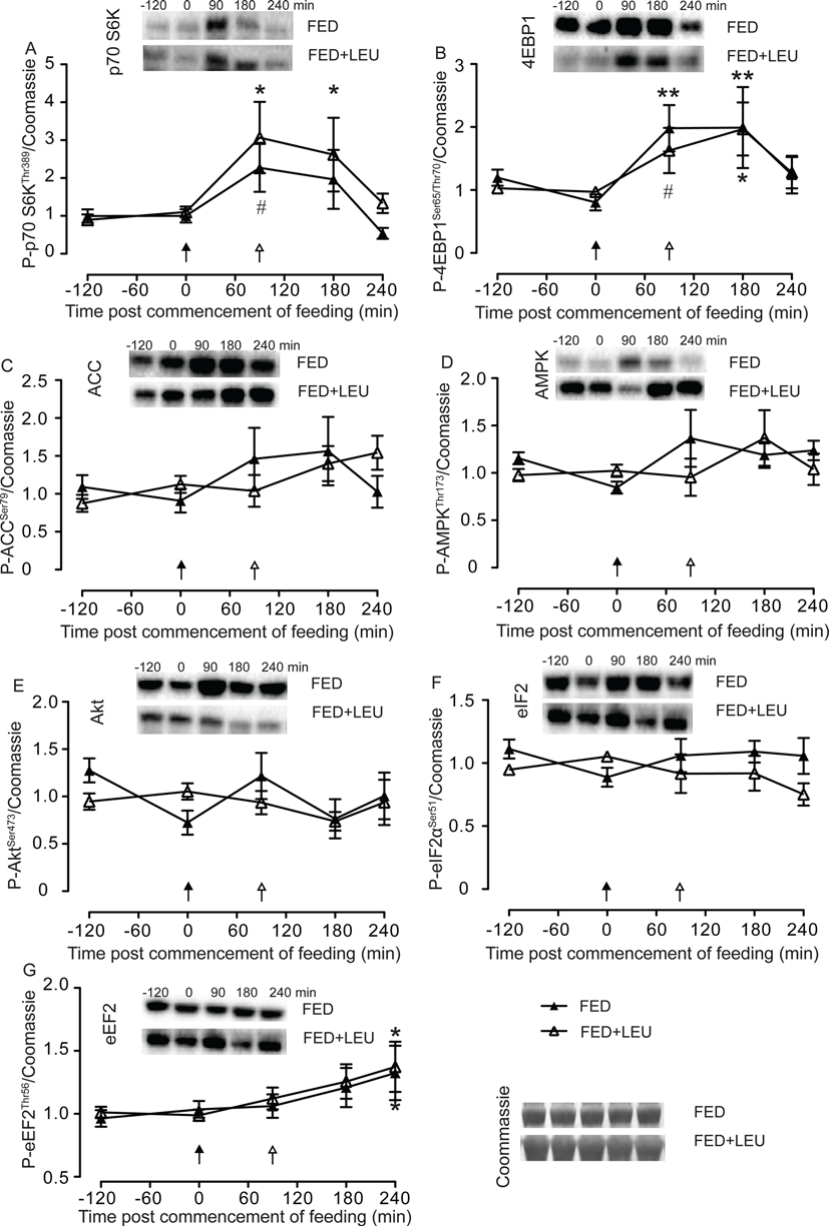
Activity within established signaling pathways Phosphorylation of ribosomal protein 70 S6 Kinase 1 (p70 S6K) Thr398 (**A**), eukaryotic translation initiation factor 4E-binding protein 1 (4EBP1) Ser65/Thr70 (**B**), acetyl Co-A carboxylase (ACCB) Ser79 (**C**), AMP-activated protein kinase (AMPK) Thr173 (**D**), protein kinase B (Akt) Ser473 (**E**), eukaryotic initiation factor 2α (eIF2α) Ser51 (**F**), and eukaryotic elongation factor 2 (eEF2) Thr56 (**G**), in older men after consumption of 15 g of mixed EAA in the absence (EAA) or presence (EAA + LEU) of a 3 g of leucine top-up. Values are means ± SE; *n*=8; ^#^ trend with *P*<0.10, *P<0.05, **P <0.01, differences from time = 0 min.

Bolus EAA feeding achieves detectable activity within the mTORC1 complex as evidenced by an increase in phosphorylation of substrates 4EBP1**^Ser65/Thr70^** (above time 0 min, or trend toward, FED *P*=0.055, 0.095, FED + LEU *P*=0.012, 0.031 at +90 and +180 min respectively) and P-p70 S6K^Thr389^ (above time 0 min, or trend toward, FED *P*=0.008, 0.079, FED + LEU *P*=0.007, 0.02 at +90 and +180 min respectively). All returned to fasting levels by 240 min. Leucine top-up failed to achieve detectable prolongation of mTORC1 activity with no difference between treatment arms (Figure 4).

EAA feeding induced phosphorylation of P-eEF2 late in the experimental period (+0.38, +0.36; *P*=0.007, *P*=0.09; 0 versus 240 min; FED, FED + LEU respectively, NS between groups, effect size at 240 min 0.079). Significant changes from fasting were not detected in phosphorylation of P-ACC^Ser79^, P-Akt^Ser473^, P-AMPK^Thr173^, nor P-eIF2α^Ser51^, with no differences detected between arms.

### Intramuscular high-energy phosphates

**Figure 5:**
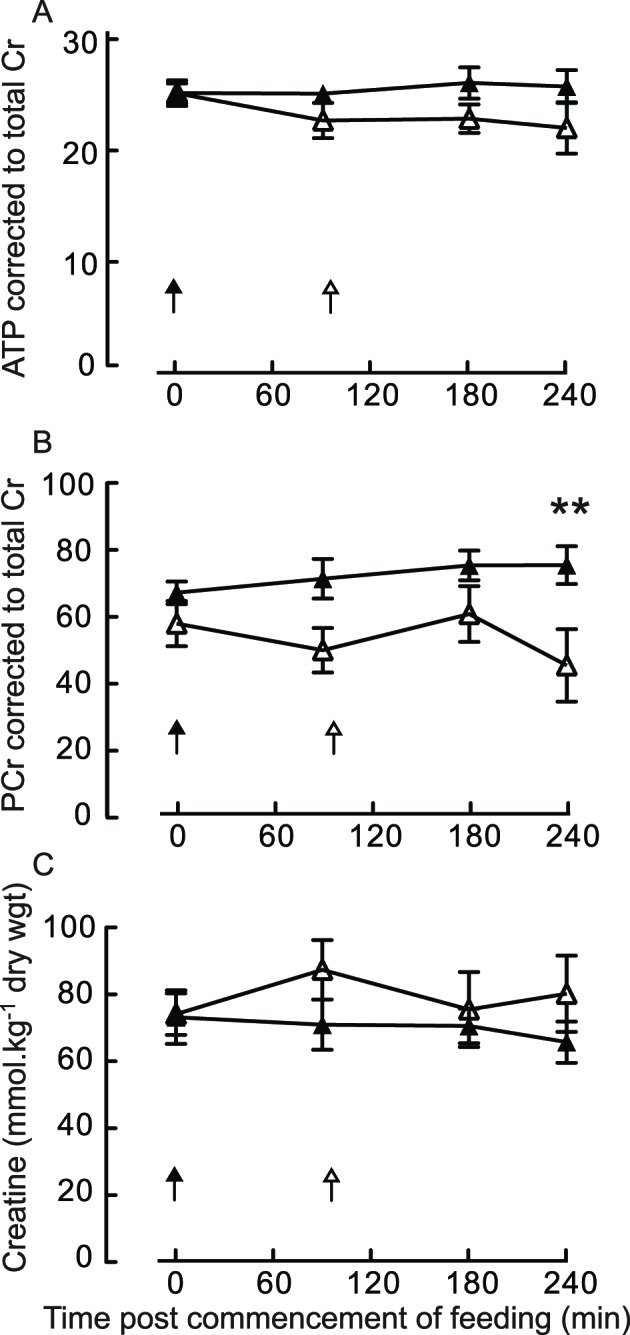
Intramuscular high energy phosphates Muscle ATP (**A**), phosphorylocreatine (PCr, **B**), and creatine (**C**) in older men after consumption of 15 g of mixed EAA in the absence (EAA) and presence (EAA + LEU) of a 3 g of leucine top-up after 90 min. All NS versus fasting. Values normalized to muscle total creatine to account for nonmuscle constituents ** P<0.01 between regimens.

Muscle PCr content was similar between feeding regimens across the fasting and early to mid-postprandial periods. However, late in the postprandial period they diverged (FED + LEU PCr 40% versus FED, *P*=0.007, effect size 2.0). Muscle creatine and ATP contents remained unchanged across the study period and were similar between feeding regimens (Figure 5).

## Discussion

An accumulating body of evidence demonstrates that a modest amount of dietary protein can achieve a maximal MPS response. The amount of myofibrillar protein accrued after such a meal is in part directed by the onset of a “muscle-full” state, of postabsorptive refractoriness to nutritional stimulation [31]. The mechanisms that underlie the onset of the muscle-full state have not been elucidated, hampering the development of interventions that could delay the onset of the muscle-full state, which may in turn help to build or maintain muscle mass. Research in this area remains current with much interest surrounding the potential of pharmaconutrients, e.g. leucine, to boost the anabolic effectiveness of feeding, especially in older populations and in those at risk of sarcopenia [32].

Here, we studied a cohort of older men in the absence and presence of a “late” postprandial period leucine top-up. As expected, provision of 15 g of EAA led to sustained hyperaminoacidemia and stimulated MPS until the onset of a “muscle-full” state 180–240 min after feeding — consistent with a fed-state anabolic window of ∼3 h [5]. Moreover, as would be expected, 3 g of leucine top-up, 90 min after feeding altered plasma aminoacidemia profiles, increasing the area under the curve for both total EAA and leucine. Nonetheless, despite enhancement of leucinemia, the temporal profile of MPS was identical between groups. By our demonstrating identical MPS responses to feeding with and without a leucine top-up in the late postprandial period, the present study has an important applied message, which challenges the notion of any benefit of supplementary leucine with, or shortly after, meals of adequate protein content. Thus, supplements of leucine should be guided in between meals or to meals containing suboptimal protein levels or quality, as we previously demonstrated in a study in older men wherein we were able to stimulate muscle anabolism when participants were given leucine in addition to 10 g of protein, following a bout of resistance exercise [33]. Detailed discussion of what should be considered an optimal protein content for a meal is beyond the scope of this paper as this will vary between people and with activity and may demand consideration of (E)AA makeup and digestion/absorption profile. That said, data from healthy, rested, fasted humans suggest that a maximal muscle protein synthetic response can be achieved by ~10 g of an appropriate mix of EAA whether delivered in as dietary protein (e.g. 113 g of beef) [3] or by oral EAA ingestion [2].

We also sought to investigate links between leucine top-ups and the molecular signals controlling MPS. In doing so, we revealed that mTORC1-related signals showed a similar response to that of MPS — albeit consistent with that which we have previously shown, anabolic signaling (i.e. mTORC1 substrates, p70S6K1, and 4EBP1) likely outlasted MPS responses, i.e. MPS was at baseline 3–4 h despite signals remaining elevated toward peak at 3 h [5,34]. These data support our MPS data in illustrating that the signals controlling increased MPS in response to nutrition are also refractory to leucine stimulation. We also measured the phosphorylation of AMPK, its substrate ACCβ and eEF2, since it had been previously reported that energy stress caused by ATP-consuming processes associated with MPS (i.e. AA transport, tRNA-aminoacylation, and translation/elongation stimulated by EAA ingestion) induced a state of ATP depletion leading to a concomitant rise in the AMP/ATP ratio and an ensuing induction of inhibitory AMPK-eEE2 signaling [23,35]. In contrast with this previous study in rodents [23] we find here, and in other similar feeding studies [5], no increases in AMPK-related signaling in either group. Moreover, in measuring ATP:PCr as a sensitive proxy of cellular energy stress [36], we failed to observe a robust induction of stress in mitochondrial energy delivery by feeding — likely ruling this out as a major facet of muscle-full in adult humans. That being said, we did observe an induction of eEF2 phosphorylation in both groups which rose to significance by 240 min; since eEF2 phosphorylation puts the brakes on mRNA translation via inhibiting A–P site translocation of ribosomes [37], this could represent an important mediator of the muscle-full state (albeit not via AMPK-signaling pathways), since eEF2 is known to be sensitive to nutrient deprivation, i.e. eEF2 is up-regulated to suppress MPS and switch off energy consuming processes. The phosphorylation of eEF2 is regulated by eEF2 kinase, which is under the regulation of AMPK [38], MAPK [39] mTOR [40], and calcium signaling related pathways [37]. Further work is needed to define the upstream kinase(s) responsible for the inhibitory induction of eEF2 phosphorylation in response to feeding, and moreover, studies with sufficient temporal definition and/or of a mechanistic nature, to determine a true role for eEF2 in regulating the muscle-full state. This is especially important since a statistically significant induction of eEF2 was evident only at 240 min, long after MPS had returned to a postabsorptive values.

Our present findings in relation to energy stress (namely the lack of) are in contrast with previous studies in rats, where the muscle-full state [23,41] appears to represent less profound refractoriness to stimulation and may be overcome by either provision of leucine or carbohydrate top-ups [23]. These differences may reflect differences between basal nutritional patterns (i.e. rats fed 80% of what they would ingest if fed *ad libitum* may represent a chronically restricted model when compared with healthy community dwelling senior human volunteers); between experimental protocols (i.e. rats fed mixed meals versus humans consuming mixed EAA isolate); between species (rat MPS have been shown to exceed human MPS by a factor of five or more [42]); and, most plausibly, differences between growing animals and weight-stable adult humans. Indeed, the very existence of an analogous muscle-full state in neonatal pigs is dubious, with an apparent unlimited stimulation of mTORC1 signaling and MPS provided sustained leucine/ EAA availability [43].

Potential limitations in the present study include avoidable variability that has been introduced by our comparison of two age-, weight-, and BMI-matched groups of eight and eight volunteers rather than undertaking interval studies on each volunteer in a cross-over paradigm. While cross-over studies provide theoretical benefit, the practical challenges of recruitment and completion of two lengthy and invasive acute studies with stable isotope tracers and multiple muscle biopsies in healthy volunteers lead the authors (and their research ethics committee) to choose to employ independent groups as described. Nonetheless, the authors conclude the present study questions the theoretical benefit, to healthy older humans, of leucine supplementation in close proximity to meals containing “adequate” protein, i.e. supplements may be most likely to be effective when taken in between meals, perhaps in the form of low dose EAA mixtures, rather than leucine alone; the efficacy of which may be limited in the absence of exogenous EAA to promote whole body and skeletal muscle net balance. Finally, given the absence of overt energy depletion, further work is needed in humans to determine the signals regulating the onset of the muscle-full state.

## Clinical perspectives

- The present study was undertaken to assess the response of human skeletal muscle to leucine top-ups after maximal essential amino acid feeding; rat studies have suggested it may promote muscle protein synthesis.
- Given after adequate doses of essential amino acids, oral leucine changes neither skeletal muscle anabolic signaling nor protein synthesis.
- These findings challenge the benefit, in healthy older people, of leucine supplements in close proximity meals of adequate protein/essential amino acids content; leucine supplements should be taken outside of meals, or with meals containing suboptimal protein. The duration of and mechanisms underlying this refractory period require further investigation.

## Abbreviations

AA: amino acid
AMPK: AMP-activated protein kinase
AUC: area under the curve
BCAA: branched-chain amino acid
EAA: essential amino acid
eEF2: eukaryotic elongation factor 2
FSR: fractional synthetic rate
MPS: muscle protein synthesis
NEAA: nonessential amino acid
PCr: phosphorylocreatine
